# Combining perineural invasion with staging improve the prognostic accuracy in colorectal cancer: a retrospective cohort study

**DOI:** 10.1186/s12885-023-11114-8

**Published:** 2023-07-18

**Authors:** Bin Zhang, Yanyun Lin, Chao Wang, Zexian Chen, Tianze Huang, Hao Chen, Guannan Wang, Ping Lan, Xiaowen He, Xiaosheng He

**Affiliations:** 1 Department of Colorectal Surgery, The Sixth Affiliated Hospital, Sun Yat-Sen University, Guangzhou, 510655 Guangdong China; 2 Department of General Surgery, The Sixth Affiliated Hospital, Sun Yat-Sen University, Guangzhou, 510655 Guangdong China; 3 Guangdong Provincial Key Laboratory of Colorectal and Pelvic Floor Diseases, The Sixth Affiliated Hospital, Sun Yat-Sen University, Guangzhou, 510655 Guangdong China; 4 Department of Pathology, The Sixth Affiliated Hospital, Sun Yat-Sen University, Guangzhou, 510655 Guangdong China

**Keywords:** Perineural invasion, Colorectal cancer, Survival, Tumor stage

## Abstract

**Background:**

Current guidelines only propose the importance of perineural invasion(PNI) on prognosis in stage II colon cancer. However, the prognostic value of PNI in other stages of colorectal cancer (CRC) is ambiguous.

**Methods:**

This single-center retrospective cohort study included 3485 CRC patients who underwent primary colorectal resection between January 2013 and December 2016 at the Sixth Affiliated Hospital of Sun Yat-sen University. Associations of PNI with overall survival (OS) and disease-free survival (DFS) were evaluated using multivariable Cox proportional hazards regression models. In addition, interaction analyses were performed to explore the prognostic effects of PNI in different clinical subgroups.

**Results:**

After median follow-up of 61.9 months, we found PNI was associated with poorer OS (adjusted hazard ratio [aHR], 1.290; 95% CI, 1.087–1.531) and DFS (aHR, 1.397; 95% CI, 1.207–1.617), irrespective of tumor stage. Interestingly, the weight of PNI was found second only to incomplete resection in the nomogram for risk factors of OS and DFS in stage II CRC patients. Moreover, OS and DFS were insignificantly different between stage II patients with PNI and stage III patients (both *P* > 0.05). PNI was found to be an independent prognostic factor of DFS in stage III CRC (aHR: 1.514; 95% CI, 1.211–1.892) as well. Finally, the adverse effect of PNI on OS was more significant in female, early-onset, and diabetes-negative patients than in their counterparts (interaction *P* = 0.0213, 0.0280, and 0.0186, respectively).

**Conclusion:**

PNI was an important prognostic factor in CRC, more than in stage II. The survival of patients with stage II combined with perineural invasion is similar with those with stage III. PNI in stage III CRC also suggests a worse survival.

**Supplementary Information:**

The online version contains supplementary material available at 10.1186/s12885-023-11114-8.

## Introduction

Colorectal cancer (CRC) is the third most frequently diagnosed cancer and the third most common cause of cancer-related deaths in the USA [1]. Meanwhile, new cases and deaths from CRC are ranked third among all malignant tumors in China, and the incidence rate and mortality of CRC in China are increasing every year [2]. The tumor-node-metastasis (TNM) classification system is the current standard for clinical prediction of survival and recurrence in CRC. In addition, several additional risk factors are used to further stratify the risk [3, 4]. To some extent, the use of these additional stratification factors suggests that the TNM system may need further improvement and supplementation. One of these stratification factors is perineural invasion (PNI). However, current guidelines only propose the importance of the PNI in stage II CRC. The prognostic value of PNI in other stages of CRC is ambiguous [5–7].

PNI is the invasion of nerves by cancer cells [8]. Several previous studies have suggested that PNI is a potential pathway for cancer cell dissemination and metastasis in the same manner as vascular and lymphatic channels [9–11]. The average detection rate of perineural invasion in CRC is 17%, ranging from 8 to 42% [12]. Previous studies have suggested that PNI may assisted in selecting patients with stage II colon cancer who could potentially benefit from adjuvant therapy [13–15], and this reminds us of the potential clinical value of PNI. However, the risk factors of PNI remain unclear. Previous studies have found that predictors of CRC with PNI include lymphovascular invasion, poor tumor differentiation, and elevated CEA levels [5, 16]. Notably, these were retrospective studies with a sample size of less than 1000. In addition, they did not explore other risk factors, such as age, since early-onset colorectal cancer (EOCRC) has aroused widespread attention.

As with unclear risk factors, the relationship between PNI and CRC prognosis remains controversial. Hu et al. reported that PNI is not an independent poor prognostic factor in patients with CRC [5]. Nevertheless, many studies have shown that PNI is a prognostic factor for non-metastatic CRC [7, 12, 13, 17–20]. Recently, some colleagues found that PNI promotes cancer progression [21, 22], suggesting that PNI might be a new metastatic spread of CRC, independent of lymphatic or vascular metastasis. In addition, there is another hypothesis that PNI may be the source of tumor deposits, which may help to improve the staging of colon cancer [23, 24]. Although the PNI has shown increasing importance, the current guidelines only purpose practical value in stage II CRC. We investigated whether PNI has influence on the prognosis of stage III CRC and found perineural invasion in stage III colorectal cancer suggests a worse survival. These indicated combined nerve-targeted therapy with chemotherapy may improve the prognosis of stage III CRC patients with PNI.

In this context, we aimed to describe PNI features and clarify the prognostic value of the PNI in CRC, especially in different tumor stages.

## Materials and methods

### Study design and patients

This single-center retrospective study was conducted at the Sixth Affiliated Hospital of Sun Yat-sen University (Guangzhou, China). Patients with CRC who underwent surgical resection of primary colorectal lesions between January 2013 and December 2016 were enrolled in the study. The inclusion criteria were as follows: (i) histological diagnosis of CRC and (ii) resection of the primary colorectal lesions. The exclusion criteria were as follows: (i) concurrent neoadjuvant therapy with pathologic complete response, (ii) no follow-up information, and (iii) insufficient clinical and pathological information. This study was approved by the Ethics Committee of the Sixth Affiliated Hospital of Sun Yat-sen University (2021ZSLYEC-542).

### Data collection and follow-up

All demographic, clinical, operative, and postoperative data were retrieved from the Colorectal Cancer Database of the Sixth Affiliated Hospital of Sun Yat-sen University. After discharge from the hospital, patients were followed up through re-examinations in the outpatient clinic and by telephone until mortality due to any reason or loss of follow-up. The detailed procedure was as follows: following completion of the treatment, follow-up studies were conducted once every three months in the three years and then once every six months for five years, and finally once a year after 5 years, as recommended in the CSCO guidelines [3]. Each follow-up study included medical history, physical examination, routine blood tests, comprehensive biochemical examinations. Thoracic-abdominal-pelvic CT scans were scheduled every 6 to 12 months after surgery for a total of 5 years, and colonoscopy was scheduled 1 year after surgery and repeated in 1 to 3 years. At the same time, we will follow up the patient's condition by telephone every 6 months.

### Study definitions

Pathology reports from all patients were reviewed for the presence of PNI. CRC in patients younger than 50 years of age was defined as EOCRC. The originating tumor proximal to the splenic flexure was classified as right-sided, while tumors arising in the splenic flexure to 15 cm of the anal verge were classified as left-sided; rectal cancers were defined as less than 15 cm from the anal verge. Overall survival (OS) was defined as the time from surgery to death from any cause. Disease-free survival (DFS) was defined as the time interval between surgery and the date of imaging/endoscopic testing, revealing the presence of recurrence or death due to any cause. Recurrence was defined based on pathological, radiological, and clinical examinations. Local recurrence was defined as tumor recurrence in the local area or nearby lymphatic flow area of the surgical operation and adjacent organs, whereas tumors at nonregional sites, such as the liver or lung, were considered distal recurrences.

### Statistical analysis

The data for this analysis were frozen in April 2021. The baseline characteristics were compared using the χ2 test. Multivariable analysis of the factors predicting CRC with PNI was performed using a logistic regression model. The 5 year OS and DFS probabilities of stage II CRC were estimated using a nomogram. Survival analysis was performed using the Kaplan–Meier method and Cox proportional hazard models. In addition, we applied a two-step method to evaluate the association between the baseline characteristics and OS and DFS. All variables were assessed using univariable Cox analyses at first, and then those parameters with *P* values < 0.05 were entered into a final multivariable Cox regression model. All statistical analyses were performed using the SPSS software (version 22.0; IBM, Armonk, NY, USA) and R software version 4.0.2 (The R Foundation for Statistical Computing, Vienna, Austria; www.r-project.org). All statistical tests were performed on two sides and *P*-value < 0.05 were identified as statistically significant.

## Results

### Patient characteristics and correlation between PNI and clinicopathological parameters

A total of 3485 patients were included, with 439 (12.6%) PNI-positive tumors and 3046 (87.4%) PNI-negative tumors (Figure S1). The baseline characteristics stratified by the presence or absence of PNI are summarized in Table 1. The incidence of PNI was higher in early-onset colorectal cancer (EOCRC) (15.8% of all EOCRC cases versus 11.5% of late-onset colorectal cancer(LOCRC), *P* = 0.0010). PNI was strongly correlated with colon cancer (*P* = 0.0009). In addition, patients with PNI were more likely to have elevated CEA, T3/4, N + , and M1, higher AJCC cancer stage, poor differentiation, lymphovascular invasion, pMMR, and incomplete resection (*P* < 0.0001). Besides, the PNI was negatively correlated with hypertension, diabetes, and BMI (*P* = 0.0399, *P* = 0.0120, and *P* = 0.0096, respectively).

**Table 1:** Baseline characteristics

	**PNI**		**Total (*N* = 3485)**	***P* value**
	No(*N* = 3046)	Yes(*N* = 439)		
**Gender, n(%)**				0.3240
Female	1181(38.8)	181(41.2)	1362(39.1)	
Male	1865(61.2)	258(58.8)	2123(60.9)	
**Age, n(%)**				0.0010
≥ 50	2329(76.5)	304(69.2)	2633(75.6)	
< 50	717(23.5)	135(30.8)	852(24.4)	
**Family history, n(%)**				0.9960
No	2949(96.8)	425(96.8)	3374(96.8)	
Yes	97(3.2)	14(3.2)	111(3.2)	
**Hypertension, n(%)**				0.0399
No	2563(84.1)	386(87.9)	2949(84.6)	
Yes	483(15.9)	53(12.1)	536(15.4)	
**Diabetes, n(%)**				0.0120
No	2796(91.8)	418(95.2)	3214(92.2)	
Yes	250(8.2)	21(4.8)	271(7.8)	
**BMI, n(%)**				0.0096
≤ 24	2061(67.7)	324(73.8)	2385(68.4)	
> 24	985(32.3)	115(26.2)	1100(31.6)	
**CEA, n(%)**				< 0.0001
≤ 5	1991(65.4)	219(49.9)	2210(63.4)	
> 5	1055(34.6)	220(50.1)	1275(36.6)	
**Tumor location, n(%)**				0.0009
Colon	1461(48.0)	251(57.2)	1712(49.1)	
Rectum	1400(46.0)	161(36.7)	1561(44.8)	
Other	185(6.1)	27(6.2)	212(6.1)	
**T, n(%)**				< 0.0001
T1/2	662(21.7)	9(2.1)	671(19.3)	
T3/4	2384(78.3)	430(97.9)	2814(80.7)	
**N, n(%)**				< 0.0001
N0	1912(62.8)	112(25.5)	2024(58.1)	
N1	838(27.5)	205(46.7)	1043(29.9)	
N2	296(9.7)	122(27.8)	418(12.0)	
**M, n(%)**				< 0.0001
M0	2660(87.3)	299(68.1)	2959(84.9)	
M1	386(12.7)	140(31.9)	526(15.1)	
**Stage, n(%)**				< 0.0001
I	543(17.8)	4(0.9)	547(15.7)	
II	1247(40.9)	82(18.7)	1329(38.1)	
III	870(28.6)	213(48.5)	1083(31.1)	
IV	386(12.7)	140(31.9)	526(15.1)	
**Differentiation, n(%)**				< 0.0001
Poor	136(4.5)	38(8.7)	174(5.0)	
Median	1915(62.9)	313(71.3)	2228(63.9)	
Well	827(27.2)	64(14.6)	891(25.6)	
Mucinous adenocarcinoma	156(5.1)	20(4.6)	176(5.1)	
Other	12(0.4)	4(0.9)	16(0.5)	
**Lymphovascular invasion, n(%) n(n(%)**				< 0.0001
No	2794(91.7)	324(73.8)	3118(89.5)	
Yes	252(8.3)	115(26.2)	367(10.5)	
**MMR status, n(%)**				< 0.0001
pMMR	2786(91.5)	430(97.9)	3216(92.3)	
dMMR	260(8.5)	9(2.1)	269(7.7)	
**Resection grade, n(%)**				< 0.0001
R0	2705(88.8)	322(73.3)	3027(86.9)	
R1	22(0.7)	6(1.4)	28(0.8)	
R2	319(10.5)	111(25.3)	430(12.3)	
**Complication, n(%)**				0.5865
No	2658(87.3)	379(86.3)	3037(87.1)	
Yes	388(12.7)	60(13.7)	448(12.9)	

According to the multivariable logistic regression analysis (Table S1), EOCRC was independent risk factors of PNI (OR, 1.391; 95% CI, 1.088–1.780; *P* = 0.0085), while dMMR was independent protective factors of PNI (OR, 0.220; 95% CI, 0.110–0.442; *P* < 0.0001, respectively).Moreover, rectal cancer, T3/4, N + , M1, and lymphovascular invasion were also independent risk factors for PNI.

### Prognostic value of the presence of perineural invasion

The median overall follow-up was 61.9 months. The 5-year OS and 5-year DFS in patients with or without PNI were 55.1%, 77.5%, 37.6%, and 68.2%, respectively (Figure S2). The univariate analysis (Table 2 and Table S2) showed that patients with PNI had a poorer OS (unadjusted hazard ratio (HR), 2.322; 95% CI, 1.977–2.727; *P* < 0.0001). According to the multivariable analysis, including parameters with *P* values < 0.05, from univariate analysis (Table 2 and Table S2), PNI was an independent predictor of OS (aHR, 1.290; 95% CI, 1.087–1.531; *P* = 0.0035). Other independent predictors of OS included male sex, LOCRC, hypertension, diabetes, BMI ≤ 24, elevated CEA, T3/4, N + , M1, poor differentiation, lymphovascular invasion, pMMR, incomplete resection, and complications. After separating colon and rectal cancer (Table 3), patients with PNI still had a poorer OS in both colon cancer (HR, 2.142; 95% CI, 1.722–2.666; *P* < 0.0001) and rectal cancer (HR, 2.549; 95% CI, 1.961–3.313; *P* < 0.0001). According to the multivariable analysis, PNI was an independent predictor of OS in rectal cancer (aHR, 1.356; 95% CI, 1.024–1.794; *P* = 0.0334). Similar results was showed in non-metastatic CRC after removal of stage IV colorectal cancer (Table 3).

**Table 2:** Univariate and multivariate cox models evaluated the effect of perineural invasion on OS and DFS

	**Univariate Cox model**		**Multivariate Cox model**	
	**Hazard Ratio**	***P*-value**	**Hazard Ratio**	***P*-value**
	**(95% CI)**		**(95% CI)**	
**OS: PNI**^a^				
No				
Yes	2.322(1.977–2.727)	< 0.0001	1.290(1.087–1.531)	0.0035
**DFS: PNI**^b^				
No				
Yes	2.495(2.177–2.859)	< 0.0001	1.397(1.207–1.617)	< 0.0001
**OS in stage III CRC: PNI**^**c**^				
No				
Yes	1.354(1.034–1.772)	0.0274	1.235(0.934–1.633)	0.1389
**DFS in stage III CRC: PNI**^**c**^				
No				
Yes	1.702(1.375–2.107)	< 0.0001	1.514(1.211–1.892)	0.0003

Parameters with *P* values < 0.05 in the univariate Cox model were then entered into a final multivariable Cox regression model. Entire tables are shown in the supplementary data

^a^Adjusted for PNI, sex, age, family history of colorectal cancer, hypertension, diabetes, BMI, CEA, T, N,M, differentiation, lymphovascular invasion, MMR status, resection grade, and complications

^b^Adjusted for PNI, sex, age, hypertension, diabetes, BMI, CEA, tumor location, T, N,M, differentiation, lymphovascular invasion, MMR status, resection grade, and complications

^c^Adjusted for PNI, age, hypertension, diabetes, CEA, T, N, differentiation, lymphovascular invasion, resection grade, and complications

**Table 3:** Univariate and multivariable cox models evaluated the effect of perineural invasion on OS and DFS in rectal cancer, colon cancer and non-metastatic CRC

	**Univariate Cox model**		**Multivariable Cox model**	
	**Hazard Ratio**	***P*-value**	**Hazard Ratio**	***P*-value**
	**(95% CI)**		**(95% CI)**	
**OS in colon cancer: PNI**^a^				
No				
Yes	2.142(1.722–2.666)	< 0.0001	1.236(0.977–1.564)	0.0768
**OS in rectal cancer: PNI**^a^				
No				
Yes	2.549(1.961–3.313)	< 0.0001	1.356(1.024–1.794)	0.0334
**DFS in colon cancer: PNI**^b^				
No				
Yes	2.451(2.031–2.957)	< 0.0001	1.414(1.153–1.734)	0.0009
**DFS in rectal cancer: PNI**^b^				
No				
Yes	2.586(2.081–3.215)	< 0.0001	1.400(1.107–1.770)	0.0050
**OS in non-metastatic CRC: PNI**^**c**^				
No				
Yes	2.137(1.710–2.670)	< 0.0001	1.447(1.140–1.837)	0.0024
**DFS in non-metastatic CRC: PNI**^**d**^				
No				
Yes	2.513(2.106–2.999)	< 0.0001	1.662(1.373–2.012)	< 0.0001

^a^Adjusted for PNI, sex, age, family history of colorectal cancer, hypertension, diabetes, BMI, CEA, T, N,M, differentiation, lymphovascular invasion, MMR status, resection grade, and complications

^b^Adjusted for PNI, sex, age, hypertension, diabetes, BMI, CEA, T, N,M, differentiation, lymphovascular invasion, MMR status, resection grade, and complications

^c^Adjusted for PNI, sex, age, hypertension, diabetes, CEA, T, N, differentiation, lymphovascular invasion, MMR status, resection grade, and complications

^d^Adjusted for PNI, sex, age, hypertension, diabetes, BMI, CEA, T, N, differentiation, lymphovascular invasion, MMR status, resection grade, and complications

Similar to the impact of PNI on OS (Table 2 and Table S3), the patients with PNI had poorer DFS (HR, 2.495; 95% CI, 2.177–2.859, *P* < 0.0001; aHR = 1.397; 95% CI 1.207–1.617; *P* < 0.0001). After separating colon and rectal cancer (Table 3), patients with PNI still had a poorer DFS in both colon cancer (HR, 2.451; 95% CI, 2.031–2.957; *P* < 0.0001; aHR, 1.414; 95% CI, 1.153–1.734; *P* = 0.0009) and rectal cancer (HR, 2.586; 95% CI, 2.081–3.215; *P* < 0.0001; aHR, 1.400; 95% CI, 1.107–1.770; *P* = 0.0050). Similar results was shown in non-metastatic CRC after removal of stage IV colorectal cancer (Table 3).

After adding adjuvant chemotherapy information in multivariable analysis (Table S6). The results showed that PNI was an independent predictor of OS (aHR, 1.302; 95% CI, 1.096–1.546; *P* = 0.0026) and DFS (aHR, 1.405; 95% CI, 1.214–1.626; *P* < 0.0001). The total clinicopathological factors used in the multivariate analysis can be seen in Table S7 and Table S8. Therefore, adding chemotherapy information in multivariable analysis does not affect prognostic value of PNI.

### Prognostic value of the presence of perineural invasion of stage II & III colorectal cancer

**Fig. 1:**
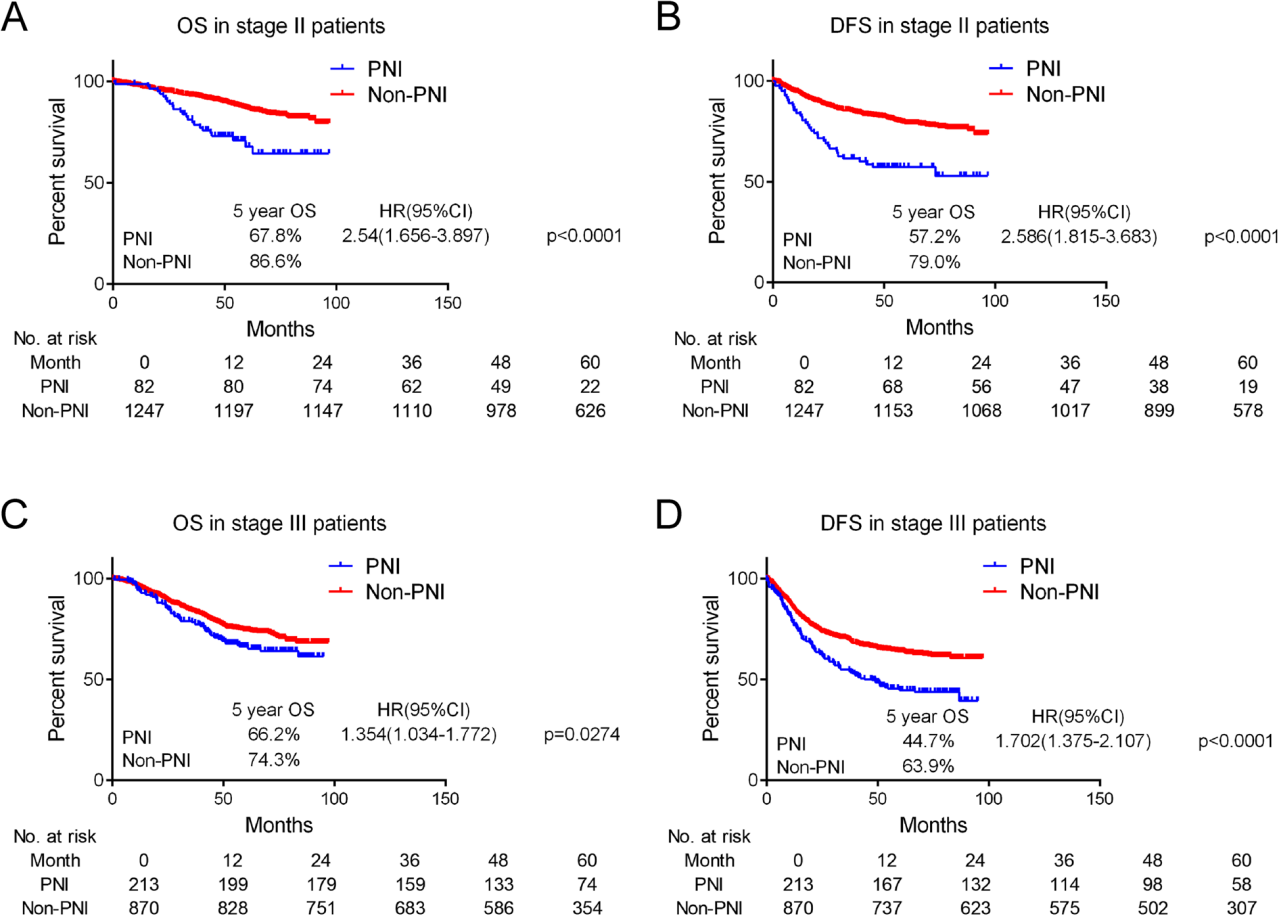
Disease-free survival and overall survival in stage II & III CRC patients according to the presence or absence of PNI. The 5-year OS in stage II patients with or without PNI were 67.8% and 86.6%, respectively (**A**). The 5-year DFS in stage II patients with or without PNI was 57.2% and 79.0%, respectively (**B**). The 5-year OS in patients with stage III patients with or without PNI were 66.2% and 74.3%, respectively (**C**). The 5-year DFS in patients with stage III patients with or without PNI were 44.7% and 63.9%, respectively (**D**)

In clinical subgroup analysis for different tumor stages, the 5-year OS and 5-year DFS in stage II patients with or without PNI were 67.8% vs. 86.6% and 57.2% vs. 79.0%, respectively (Fig. 1A and B). In addition, the 5-year OS and 5-year DFS in patients with stage III disease with or without PNI were 66.2% and 74.3%, and 44.7% and 63.9%, respectively (Fig. 1C and D).

The predictive factors of OS in univariate and multivariable analyses in stage III CRC were shown in Table 2 and Table S4. Patients with PNI had poorer OS (HR, 1.354; 95% CI, 1.034–1.772; *P* = 0.0274; aHR, 1.235; 95% CI, 0.934–1.633; *P* = 0.1389) in stage III CRC. For DFS in stage III CRC (Table 2 and Table S5), patients with PNI had poorer DFS in univariate analysis (HR, 1.702; 95% CI, 1.375–2.107; *P* < 0.0001; aHR: 1.514; 95% CI, 1.211–1.892; *P* = 0.0003). Furthermore, similar results was shown in stage III CRC After adding adjuvant chemotherapy information in multivariable analysis (Table S6).

**Fig. 2:**
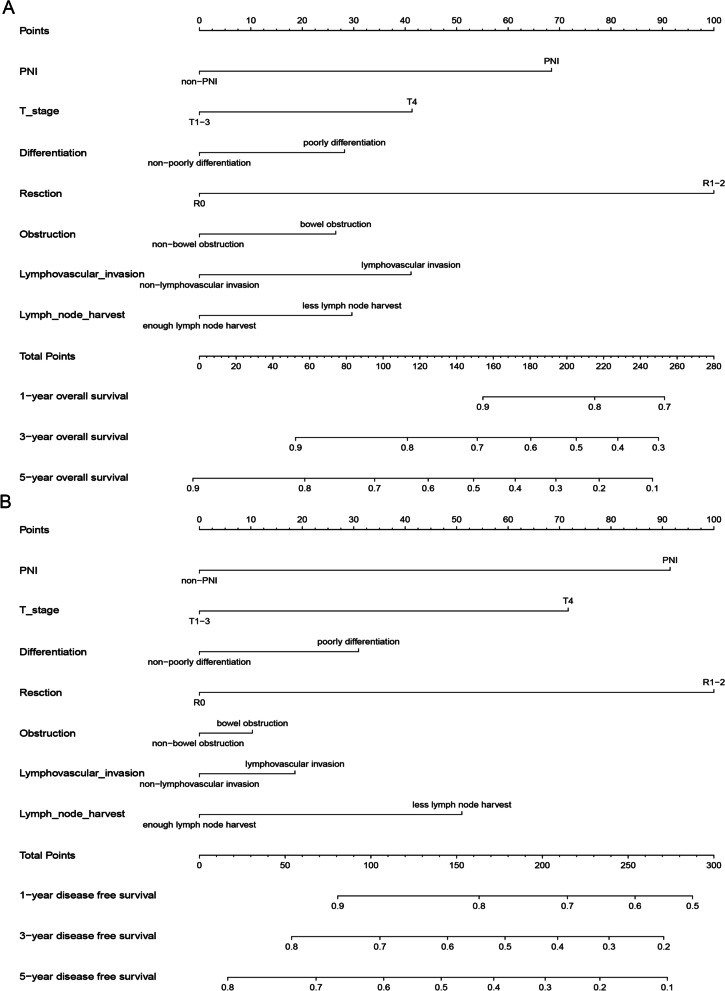
Nomograms in stage II CRC patients according to risk factor of stage II diseases. A nomogram for risk factors for OS in patients with stage II CRC indicated that the weight of PNI in stage II risk factors was second only to incomplete resection (**A**). A nomogram for risk factors for DFS in patients with stage II CRC indicated that the weight of PNI in stage II risk factors was second only to incomplete resection (**B**)

The PNI is a risk factor for stage II colon cancer [4, 14, 15]. We wondered which of these risk factors contributes most to the prognosis. The weight of PNI was found second only to incomplete resection in the nomogram for risk factors of OS and DFS in stage II CRC patients (Fig. 2A). Similar results were observed in the nomogram for risk factors for DFS (Fig. 2B).

**Fig. 3:**
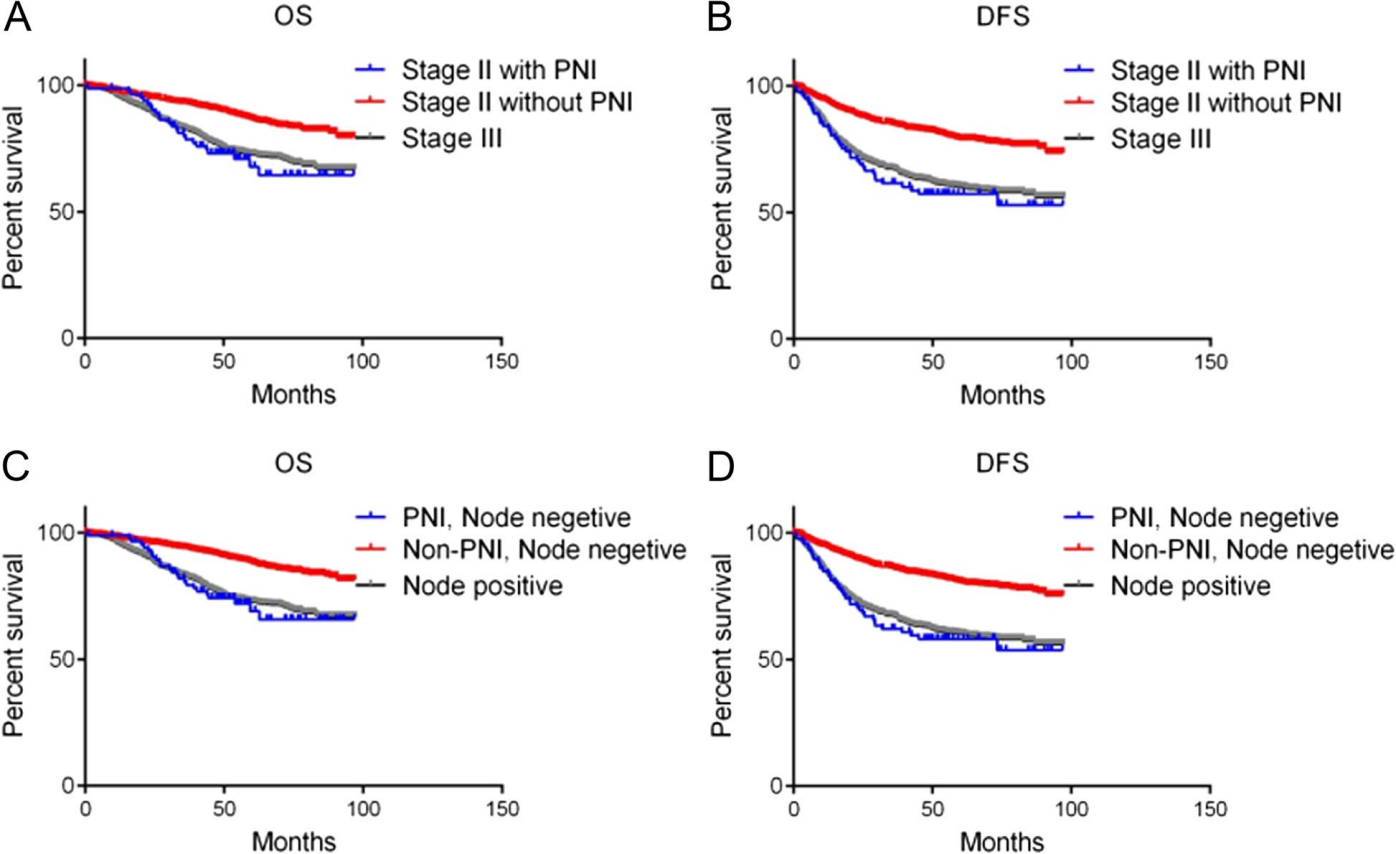
Disease-free survival and overall survival in non-metastatic CRC patients according to the presence or absence of PNI. OS and DFS were not significantly different between stage II patients with PNI and stage III patients (HR,1.126; 95% CI, 0.731–1.755 and HR,1.136; 95% CI. 0.795–1.646, respectively; **A** and **B**). After further inclusion of stage I CRC patients, OS and DFS were not significantly different between lymph node-negative patients with PNI and lymph node-positive patients(HR,1.075; 95% CI, 0.702–1.656 and HR, 1.105; 95% CI, 0.778–1.583, respectively; **C** and **D**)

Survival analysis was performed in stage II and III CRC patients to further explore the effect of PNI on non-metastatic CRC patients (Fig. 3). OS and DFS were insignificantly different between stage II patients with PNI and stage III patients (HR,1.126; 95% CI, 0.731–1.755 and HR,1.136; 95% CI. 0.795–1.646, respectively; Fig. 3A and B). After further inclusion of stage I CRC patients, OS and DFS were insignificantly different between lymph node-negative patients with PNI and lymph node-positive patients (HR,1.075; 95% CI, 0.702–1.656 and HR, 1.105; 95% CI, 0.778–1.583, respectively; Fig. 3C and D).

### The interaction analyses of perineural invasion on prognosis among clinical subgroups

**Fig. 4:**
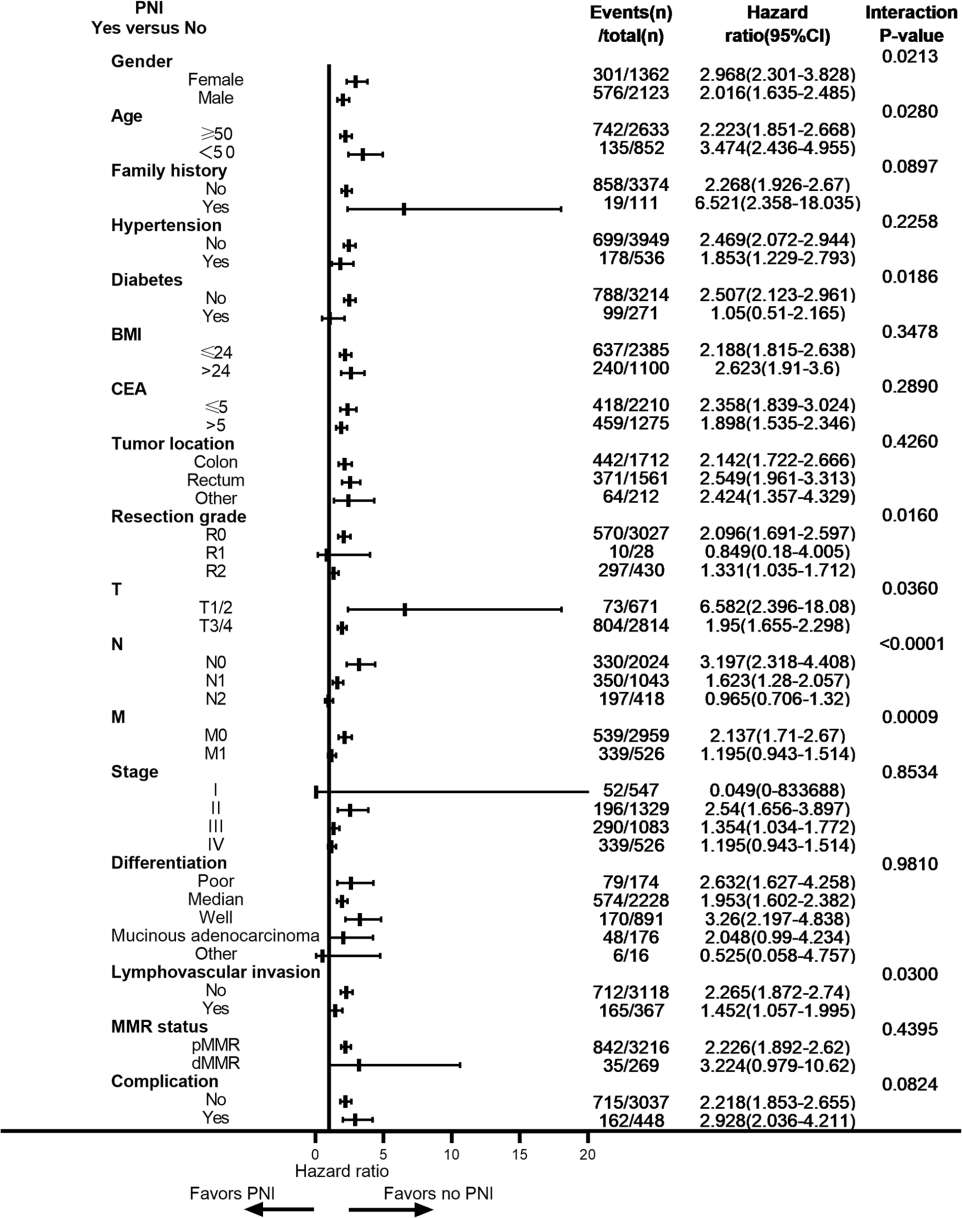
Forest plot for the effect of perineural invasion on overall survival among clinical subgroups

The adverse effect of PNI on OS was more significant in women, which suggested that PNI might be worse in women than in men (interaction *P* = 0.0213; Fig. 4). In addition, there was evidence of an interaction between PNI and age, which indicated that compared with non-PNI patients, patients with PNI had a greater impact on OS in EOCRC than in LOCRC (interaction *P* = 0.0280). Similarly, the adverse effect of PNI on OS was more significant in diabetes-negative patients, complete resection, T1/2, N0, M0, and lymphovascular invasion-negative patients than in their counterparts ( Fig. 4).

The adverse effect of PNI on DFS was more significant in female patients than in male patients (interaction *P* = 0.0384, Figure S3). Meanwhile, there was a trend of interaction on DFS, including age and diabetes (interaction *P* = 0.0913 and 0.0840, Figure S3). Finally, the adverse effect of PNI on DFS was more significant in family history, hypertension, CEA ≤ 5, complete resection, N0, M0, and lymphovascular invasion negative patients than in their counterparts (Figure S3).

## Discussion

Perineural invasion was defined as the presence of cancer cells in the nerves or surrounding or pass-through nerves, tumor cells close to the nerve and surrounding at least 33% of the nerve periphery, or tumor cells invading any of the three layers of the neurolemma structure [25]. PNI in CRC specimens was observed in 12.6% of the patients in our single-center retrospective study. Deborde et al. found that PNI induced cancer cell dispersion and invasion [10], while Demir et al. considered that PNI was associated with cancer pain. However, few studies had focused on the risk factors for PNI [5, 16, 26, 27]. Previous studies had found that predictors of CRC with PNI include lymphovascular invasion, differentiation, and elevated CEA levels. In this study, multivariate logistic regression analysis demonstrated that factors predicting CRC with PNI included EOCRC, colon cancer, and pMMR. Moreover, the adverse effects of PNI on survival were more significant in patients with EOCRC. This finding indicated that nerve dissection might be more appropriate for treating EOCRC. Colon cancer had a higher PNI rate in our study. The colon innervated by nerve fibers originating from inferior mesenteric ganglia and superior mesenteric ganglia (sympathetic efferent), while the rectum innervated by nerve fibers originating from pelvic ganglia (parasympathetic efferent) [28]. Besides, sympathetic nerves facilitate cancer progression [29]. This may mean that CRC cells are more likely to invade the sympathetic nervous system. dMMR CRCs are characterized by a high tumor mutation burden that leads to abundant mutation-derived neoantigens that trigger a robust immune response in the tumor microenvironment with tumor-infiltrating lymphocytes [30]. In pMMR CRC, it is more likely that the neural microenvironment plays a major role in PNI, rather than tumor-infiltrating lymphocytes.

Many clinical guidelines [3, 4] and studies [13, 31] suggested that PNI was a risk factor for stage II colon cancer. To explore which factor contributed most to the prognosis among these risk factors (PNI, incomplete resection, lymphovascular invasion, T4, poor differentiation, less lymph node harvest, and bowel obstruction), a nomogram for risk factors for OS and DFS in stage II CRC patients indicated that the weight of PNI was second only to incomplete resection (Fig. 2A and B). This indicated that PNI was an important risk factor for stage II CRC. However, Baxter et al. found that the impact of adjuvant chemotherapy on survival in stage II patients with PNI was uncertain [31]. As the prognosis of stage II CRC with PNI is poor and the effect of adjuvant chemotherapy is uncertain, there is an urgent need for a new nerve-targeted therapy to improve the prognosis of stage II CRC patients with PNI.

PNI is considered both a form of local progression and a form of metastasis, since nerve invasion may extend proximally to reach the central nervous system [32, 33]. In our study, OS and DFS were not significantly different between stage II patients with PNI and III patients (Fig. 3A and B). PNI was an independent prognostic factor for stage III CRC recurrence (Table 2 and Table S5). PNI may be a source of tumor deposits, which improves CRC staging [23, 24]. Therefore, PNI is a form of metastasis that is parallel to lymph node and vascular metastases. The position of the PNI during staging should be improved and nerve-targeted therapy is likely to improve prognosis of stage III CRC patients with PNI.

In our study, female patients or patients without diabetes combined with PNI had a worse prognosis than the corresponding patients. Cancer cells secrete vascular endothelial growth factor (VEGF) A and platelet-activating factor in response to estrogens, enhancing proliferation and migration [34]. These results indicate that female estrogen may promote the recurrence and metastasis of nerve-infiltrating tumor cells. Peripheral nerve injury is a common complication in diabetes patients [35]. Patients with diabetes are accompanied by more nerve injury, which may hamper the invasion of cancer cells to nerve system. The adverse effects of PNI were more significant in patients without diabetes due to the complete nerve microenvironment.

Our study has several limitations. First, this single-center retrospective study design increased recall and information bias, which may limit the outreach of the conclusion. The prognostic value of the PNI was best investigated in a randomized controlled trial. Another potential source of error is pathology reports from single institutions without a complete review, and the study did not require specific expertise in the review of PNI. The advantages of the study include the large number of patients with detailed clinicopathological information and the timing of death and recurrence. Another advantage of this study is that it contains a specific analysis of subgroups of patients with EOCRC, women, and patients without diabetes who had never been studied.

## Conclusions

In conclusion, the prognosis of stage II colorectal cancer combined with PNI is consistent with the prognosis of stage III colorectal cancer. Combining PNI with stage III colorectal cancer suggests a worse prognosis. The importance of PNI for recurrence and survival of risk factors is second only to incomplete resection in stage II CRC. EOCRC is more likely to occur with PNI, which predicts worse prognosis. Female patients or patients without diabetes combined with PNI predict a worse prognosis than the corresponding patients.

## Supplementary Information

**Additional file 1:** **Figure S1.** Flow Chart of theIncluded Participants in this Study. **Figure S2.** Overall survival andDisease-free survival stratified by the presence/absence of PNI. The 5-year OS in patients with or without PNI were 55.1% and 77.5% , respectively(Figure S2A). The 5-year DFS in patients with or without PNI were 37.6%, and 68.2%,respectively (Figure S2B). **Figure S3.** Forest plot for theeffect of perineural invasion on disease-free survival among clinical subgroups.**Table S1.** Multivariable analysis of factors predicting colorectalcancer with PNI. **Table S2. **Univariate and Multivariable cox models foroverall survival of baseline characteristics. **Table S3. **Univariate and Multivariable cox models fordisease-free survival of baseline characteristics. **Table S4. **Univariate and Multivariable cox models foroverall survival of Stage III patients. **Table S5. **Univariate and Multivariable Cox models fordisease-free survival of patientswith stage III disease. **Table S6. **Tables after adding adjuvant chemotherapy. **Table S7. **Univariate and Multivariablecox models for overall survival of baseline characteristics after adding adjuvantchemotherapy. **Table S8.** Univariate and Multivariablecox models for disease-free survival of baseline characteristics after adding adjuvantchemotherapy.

## Data Availability

The datasets used and analyzed during the current study are available from the corresponding author on reasonable request.

## Abbreviations

PNI: Perineural invasion
CRC: Colorectal cancer
DFS: Disease free survival
OS: Overall survival
HR: Hazard ratio
TNM: Tumor-node-metastasis
EOCRC: Early-onset colorectal cancer
LOCRC: Late-onset colorectal cancer
OR: Odds ratio
CI: Confidence interval
MMR: Mismatch repair
