# Supplementary material for: Combining perineural invasion with staging improve the prognostic accuracy in colorectal cancer: a retrospective cohort study
Source: BMC Cancer. 2023 Jul 18;23:675. doi: 10.1186/s12885-023-11114-8 (PMC10354907; doi:10.1186/s12885-023-11114-8)
Supplement: Supplementary file 1 — Additional file 1: Figure S1. Flow Chart of theIncluded Participants in this Study. Figure S2. Overall survival andDisease-free survival stratified by the presence/absence of PNI. The 5-year OS in patients with or without PNI were 55.1% and 77.5% , respectively(Figure S2A). The 5-year DFS in patients with or without PNI were 37.6%, and 68.2%,respectively (Figure S2B). Figure S3. Forest plot for theeffect of perineural invasion on disease-free survival among clinical subgroups.Table S1. Multivariable analysis of factors predicting colorectalcancer with PNI. Table S2. Univariate and Multivariable cox models foroverall survival of baseline characteristics. Table S3. Univariate and Multivariable cox models fordisease-free survival of baseline characteristics. Table S4. Univariate and Multivariable cox models foroverall survival of Stage III patients. Table S5. Univariate and Multivariable Cox models fordisease-free survival of patientswith stage III disease. Table S6. Tables after adding adjuvant chemotherapy. Table S7. Univariate and Multivariablecox models for overall survival of baseline characteristics after adding adjuvantchemotherapy. Table S8. Univariate and Multivariablecox models for disease-free survival of baseline characteristics after adding adjuvantchemotherapy. [file 12885_2023_11114_MOESM1_ESM.doc]

**Combining perineural invasion with staging improve the prognostic accuracy in colorectal cancer: a retrospective cohort study**

Supplementary Material

**LEGENDS FOR Figure S1-S3**

**Figure S1**. Flow Chart of the Included Participants in this Study

**Figure S2**. Overall survival and Disease-free survival stratified by the presence/absence of PNI. The 5-year OS in patients with or without PNI were 55.1% and 77.5% , respectively (Figure S2A). The 5-year DFS in patients with or without PNI were 37.6%, and 68.2%, respectively (Figure S2B).

**Figure S3**. Forest plot for the effect of perineural invasion on disease-free survival among clinical subgroups.

**Figure S1:** Flow Chart

colorectal cancer patients（n=4285）

Exclude（n=800）

- No follow-up information（n=24）
- pathologic complete response（n=209）
- Insufficient clinical information and pathologic information（n=567）

Include patients（n=3485）

PNI group（n=439）

Non-PNI group（n=3046）

**Figure S2:** Overall survival and Disease-free survival stratified by the presence/absence of PNI

A





B





**Figure S3:** Forest plot for the effect of perineural invasion on disease-free survival among clinical subgroups.


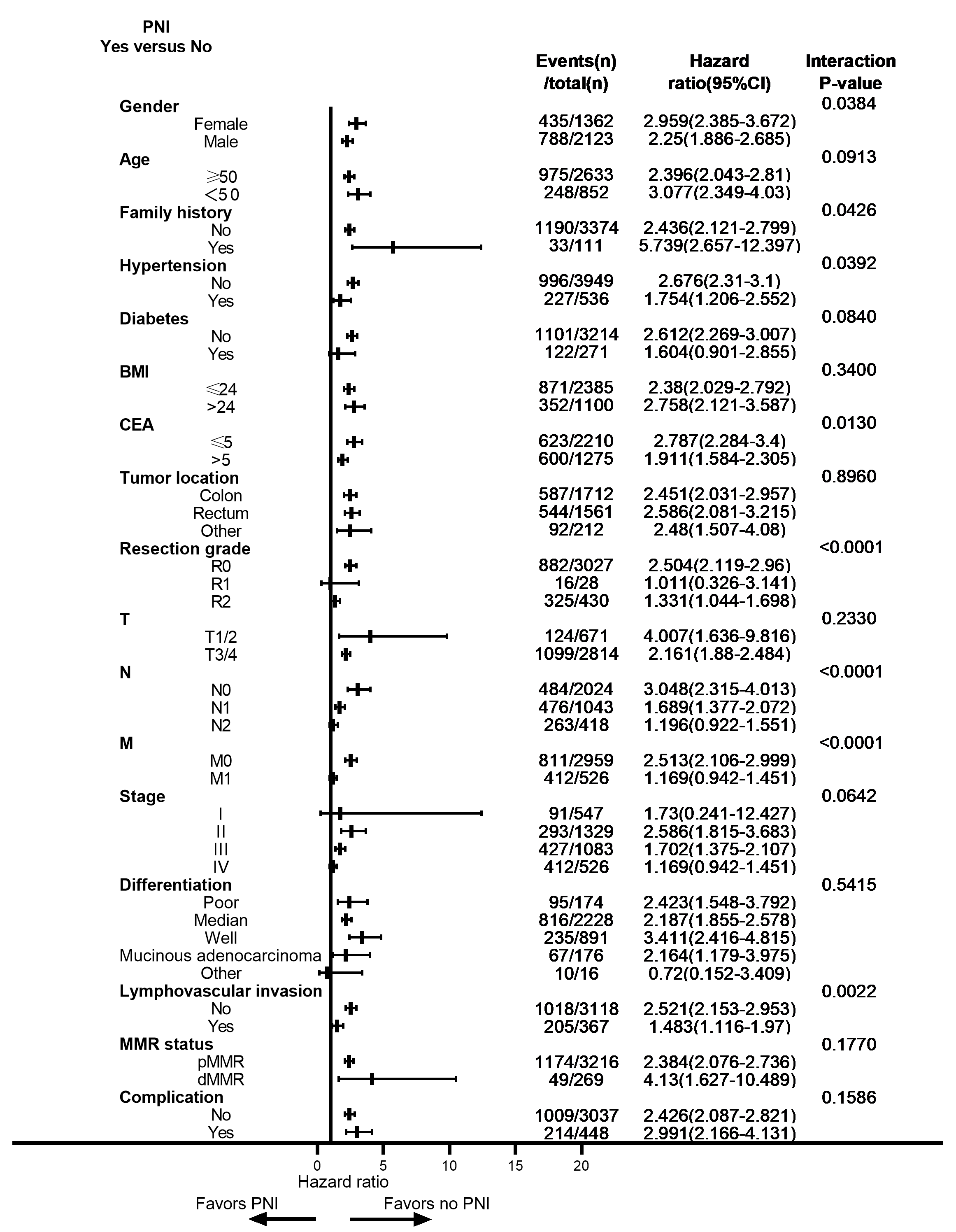


**Table S1** Multivariable analysis of factors predicting colorectal cancer with PNI

|  | **Odds ratio** | **95% CI** | **P value** |
| --- | --- | --- | --- |
| **Age(＜50 VS ≥50)** | 1.391 | 1.088-1.78 | 0.0085 |
| **Hypertension** | 0.761 | 0.544-1.065 | 0.1110 |
| **Diabetes** | 0.614 | 0.376-1.001 | 0.0507 |
| **BMI(＞24 VS ≤24)** | 0.786 | 0.616-1.002 | 0.0521 |
| **CEA** | 1.203 | 0.957-1.512 | 0.1128 |
| **Tumor location** |  |  |  |
| Colon | 1 |  | 0.0357 |
| Rectum | 0.741 | 0.589-0.931 | 0.0100 |
| Other | 0.927 | 0.588-1.46 | 0.7429 |
| **T (T3/4 vs T1/2)** | 7.071 | 3.582-13.959 | <0.0001 |
| **N** |  |  |  |
| N0 | 1 |  | <0.0001 |
| N1 | 2.736 | 2.114-3.54 | <0.0001 |
| N2 | 3.486 | 2.527-4.809 | <0.0001 |
| **M (M1 vs M0)** | 1.589 | 1.073-2.353 | 0.0208 |
| **Differentiation** |  |  |  |
| Poor | 1 |  | 0.1767 |
| Median | 1.086 | 0.702-1.679 | 0.7108 |
| Well | 0.792 | 0.479-1.311 | 0.3648 |
| Mucinous adenocarcinoma | 0.708 | 0.373-1.345 | 0.2912 |
| Other | 1.090 | 0.315-3.769 | 0.8921 |
| **Lymphovascular invasion** | 2.079 | 1.565-2.761 | <0.0001 |
| **dMMR** | 0.220 | 0.11-0.442 | <0.0001 |
| **Resection grade** |  |  |  |
| R0 | 1 |  | 0.6623 |
| R1 | 1.576 | 0.59-4.212 | 0.3640 |
| R2 | 1.005 | 0.658-1.536 | 0.9805 |
|  |  |  |  |

All variables were assessed using the chi-square test at first in baseline characteristics, and then those parameters with P values <0.05 were entered into a final multivariable logistic regression model.

CI = confidence interval, PNI = perineural invasion,MMR = mismatch repair.

**Table S2** Univariate and Multivariable cox models for overall survival of baseline characteristics

|  | **Univariate Cox model** | | **Multivariable Cox model** | |
| --- | --- | --- | --- | --- |
|  | **Hazard Ratio** | **P-value** | **Hazard Ratio** | **P-value** |
|  | **(95% CI)** |  | **(95% CI)** |  |
| **PNI** |  |  |  |  |
| No |  |  |  |  |
| Yes | 2.322(1.977-2.727) | <0.0001 | 1.290(1.087-1.531) | 0.0035 |
| **Gender** |  |  |  |  |
| Female |  |  |  |  |
| Male | 1.27(1.105-1.46) | 0.0008 | 1.153(1.001-1.327) | 0.0479 |
| **Age** |  |  |  |  |
| ≥50 |  |  |  |  |
| ＜50 | 0.523(0.436-0.629) | <0.0001 | 0.487(0.402-0.59) | <0.0001 |
| **Family history** |  |  |  |  |
| No |  |  |  |  |
| Yes | 0.618(0.392-0.974) | 0.0380 | 0.803(0.508-1.271) | 0.3493 |
| **Hypertension** |  |  |  |  |
| No |  |  |  |  |
| Yes | 1.528(1.296-1.801) | <0.0001 | 1.214(1.02-1.445) | 0.0291 |
| **Diabetes** |  |  |  |  |
| No |  |  |  |  |
| Yes | 1.693(1.373-2.087) | <0.0001 | 1.579(1.271-1.96) | <0.0001 |
| **BMI** |  |  |  |  |
| ≤24 |  |  |  |  |
| >24 | 0.791(0.682-0.918) | 0.0020 | 0.793(0.682-0.924) | 0.0028 |
| **CEA** |  |  |  |  |
| ≤5 |  |  |  |  |
| >5 | 2.316(2.028-2.645) | <0.0001 | 1.296(1.122-1.497) | 0.0004 |
| **Tumor location** |  |  |  |  |
| Colon |  |  |  |  |
| Rectum | 0.889(0.774-1.021) | 0.0950 |  |  |
| Other | 1.206(0.928-1.568) | 0.1614 |  |  |
| **T** |  |  |  |  |
| T1/2 |  |  |  |  |
| T3/4 | 3.032(2.386-3.853) | <0.0001 | 1.581(1.228-2.035) | 0.0004 |
| **N** |  |  |  |  |
| N0 |  |  |  |  |
| N1 | 2.394(2.059-2.783) | <0.0001 | 1.578(1.343-1.854) | <0.0001 |
| N2 | 3.961(3.318-4.728) | <0.0001 | 1.983(1.624-2.42) | <0.0001 |
| **M** |  |  |  |  |
| M0 |  |  |  |  |
| M1 | 6.242(5.438-7.166) | <0.0001 | 1.973(1.545-2.519) | <0.0001 |
| **Stage** |  |  |  |  |
| I |  |  |  |  |
| II | 1.603(1.181-2.176) | 0.0025 |  |  |
| III | 3.216(2.394-4.32) | <0.0001 |  |  |
| IV | 12.746(9.508-17.087) | <0.0001 |  |  |
| **Differentiation** |  |  |  |  |
| Poor |  |  |  |  |
| Median | 0.424(0.335-0.536) | <0.0001 | 0.510(0.397-0.655) | <0.0001 |
| Well | 0.275(0.21-0.359) | <0.0001 | 0.442(0.332-0.59) | <0.0001 |
| Mucinous adenocarcinoma | 0.443(0.31-0.635) | <0.0001 | 0.583(0.404-0.841) | 0.0039 |
| Other | 0.764(0.333-1.752) | 0.5248 | 0.660(0.285-1.526) | 0.3312 |
| **Lymphovascular invasion** |  |  |  |  |
| No |  |  |  |  |
| Yes | 2.463(2.079-2.919) | <0.0001 | 1.339(1.11-1.616) | 0.0023 |
| **MMR status** |  |  |  |  |
| pMMR |  |  |  |  |
| dMMR | 0.45(0.321-0.63) | <0.0001 | 0.615(0.435-0.87) | 0.0061 |
| **Resection grade** |  |  |  |  |
| R0 |  |  |  |  |
| R1 | 2.508(1.342-4.687) | 0.0040 | 1.992(1.058-3.75) | 0.0328 |
| R2 | 7.234(6.271-8.345) | <0.0001 | 2.859(2.233-3.661) | <0.0001 |
| **Complication** |  |  |  |  |
| No |  |  |  |  |
| Yes | 1.786(1.506-2.119) | <0.0001 | 1.670(1.405-1.985) | <0.0001 |

Parameters with P values <0.05 in the univariate Cox model were then entered into a final multivariable Cox regression model.

CI = confidence interval, PNI = perineural invasion,MMR = mismatch repair.

**Table S3** Univariate and Multivariable cox models for disease-free survival of baseline characteristics

|  | **Univariate Cox model** | | **Multivariable Cox model** | |
| --- | --- | --- | --- | --- |
|  | **Hazard Ratio** | **P-value** | **Hazard Ratio** | **P-value** |
|  | **(95% CI)** |  | **(95% CI)** |  |
| **PNI** |  |  |  |  |
| No |  |  |  |  |
| Yes | 2.495(2.177-2.859) | <0.0001 | 1.397(1.207-1.617) | <0.0001 |
| **Gender** |  |  |  |  |
| Female |  |  |  |  |
| Male | 1.182(1.051-1.329) | 0.0051 | 1.091(0.969-1.228) | 0.1512 |
| **Age** |  |  |  |  |
| ≥50 |  |  |  |  |
| ＜50 | 0.756(0.657-0.869) | 0.0001 | 0.723(0.624-0.837) | <0.0001 |
| **Family history** |  |  |  |  |
| No |  |  |  |  |
| Yes | 0.792(0.56-1.119) | 0.1867 |  |  |
| **Hypertension** |  |  |  |  |
| No |  |  |  |  |
| Yes | 1.344(1.164-1.553) | 0.0001 | 1.149(0.985-1.34) | 0.0764 |
| **Diabetes** |  |  |  |  |
| No |  |  |  |  |
| Yes | 1.449(1.201-1.747) | 0.0001 | 1.406(1.158-1.706) | 0.0006 |
| **BMI** |  |  |  |  |
| ≤24 |  |  |  |  |
| >24 | 0.848(0.749-0.96) | 0.0092 | 0.869(0.766-0.986) | 0.0298 |
| **CEA** |  |  |  |  |
| ≤5 |  |  |  |  |
| >5 | 2.033(1.817-2.275) | <0.0001 | 1.243(1.1-1.405) | 0.0005 |
| **Tumor location** |  |  |  |  |
| Colon |  |  |  |  |
| Rectum | 0.994(0.885-1.117) | 0.9207 | 1.148(1.016-1.297) | 0.0263 |
| Other | 1.364(1.095-1.699) | 0.0056 | 1.317(1.054-1.644) | 0.0153 |
| **T** |  |  |  |  |
| T1/2 |  |  |  |  |
| T3/4 | 2.509(2.083-3.021) | <0.0001 | 1.499(1.229-1.828) | 0.0001 |
| **N** |  |  |  |  |
| N0 |  |  |  |  |
| N1 | 2.302(2.028-2.613) | <0.0001 | 1.584(1.383-1.813) | <0.0001 |
| N2 | 3.912(3.364-4.549) | <0.0001 | 2.111(1.78-2.504) | <0.0001 |
| **M** |  |  |  |  |
| M0 |  |  |  |  |
| M1 | 5.068(4.49-5.722) | <0.0001 | 2.83(2.309-3.469) | <0.0001 |
| **Stage** |  |  |  |  |
| I |  |  |  |  |
| II | 1.385(1.095-1.752) | 0.0067 |  |  |
| III | 2.88(2.297-3.612) | <0.0001 |  |  |
| IV | 9.174(7.298-11.532) | <0.0001 |  |  |
| **Differentiation** |  |  |  |  |
| Poor |  |  |  |  |
| Median | 0.507(0.41-0.627) | <0.0001 | 0.657(0.525-0.822) | 0.0002 |
| Well | 0.325(0.256-0.413) | <0.0001 | 0.561(0.435-0.723) | <0.0001 |
| Mucinous adenocarcinoma | 0.527(0.385-0.72) | 0.0001 | 0.699(0.508-0.961) | 0.0274 |
| Other | 1.067(0.556-2.047) | 0.8460 | 0.892(0.461-1.726) | 0.7347 |
| **Lymphovascular invasion** |  |  |  |  |
| No |  |  |  |  |
| Yes | 2.224(1.914-2.585) | <0.0001 | 1.165(0.988-1.373) | 0.0695 |
| **MMR status** |  |  |  |  |
| pMMR |  |  |  |  |
| dMMR | 0.444(0.334-0.591) | <0.0001 | 0.612(0.456-0.821) | 0.0010 |
| **Resection grade** |  |  |  |  |
| R0 |  |  |  |  |
| R1 | 2.496(1.522-4.093) | 0.0003 | 1.786(1.08-2.952) | 0.0238 |
| R2 | 4.437(3.898-5.051) | <0.0001 | 1.227(0.991-1.518) | 0.0606 |
| **Complication** |  |  |  |  |
| No |  |  |  |  |
| Yes | 1.675(1.445-1.942) | <0.0001 | 1.481(1.274-1.722) | <0.0001 |

Parameters with P values <0.05 in the univariate Cox model were then entered into a final multivariable Cox regression model.

CI = confidence interval, PNI = perineural invasion,MMR = mismatch repair.

**Table S4** Univariate and Multivariable cox models for overall survival of Stage Ⅲ patients

|  | **Univariate Cox model** | | **Multivariable Cox model** | |
| --- | --- | --- | --- | --- |
|  | **Hazard Ratio** | **P-value** | **Hazard Ratio** | **P-value** |
|  | **(95% CI)** |  | **(95% CI)** |  |
| **PNI** |  |  |  |  |
| No |  |  |  |  |
| Yes | 1.354(1.034-1.772) | 0.0274 | 1.235(0.934-1.633) | 0.1389 |
| **Gender** |  |  |  |  |
| Female |  |  |  |  |
| Male | 1.237(0.975-1.569) | 0.0804 |  |  |
| **Age** |  |  |  |  |
| ≥50 |  |  |  |  |
| ＜50 | 0.449(0.327-0.617) | <0.0001 | 0.389(0.277-0.545) | <0.0001 |
| **Family history** |  |  |  |  |
| No |  |  |  |  |
| Yes | 0.63(0.28-1.415) | 0.2628 |  |  |
| **Hypertension** |  |  |  |  |
| No |  |  |  |  |
| Yes | 1.631(1.23-2.164) | 0.0007 | 1.279(0.944-1.733) | 0.1128 |
| **Diabetes** |  |  |  |  |
| No |  |  |  |  |
| Yes | 1.801(1.253-2.589) | 0.0015 | 1.537(1.051-2.248) | 0.0267 |
| **BMI** |  |  |  |  |
| ≤24 |  |  |  |  |
| >24 | 0.823(0.638-1.062) | 0.1338 |  |  |
| **CEA** |  |  |  |  |
| ≤5 |  |  |  |  |
| >5 | 1.326(1.05-1.675) | 0.0177 | 1.158(0.909-1.475) | 0.2342 |
| **Tumor location** |  |  |  |  |
| Colon |  |  |  |  |
| Rectum | 1.226(0.965-1.557) | 0.0956 |  |  |
| Other | 1.271(0.793-2.038) | 0.3190 |  |  |
| **T** |  |  |  |  |
| T1/2 |  |  |  |  |
| T3/4 | 2.712(1.555-4.73) | 0.0004 | 2.191(1.245-3.858) | 0.0066 |
| **N** |  |  |  |  |
| N1 |  |  |  |  |
| N2 | 1.97(1.552-2.499) | <0.0001 | 1.691(1.305-2.191) | 0.0001 |
| **Differentiation** |  |  |  |  |
| Poor |  |  |  |  |
| Median | 0.437(0.305-0.627) | <0.0001 | 0.522(0.354-0.771) | 0.0011 |
| Well | 0.324(0.208-0.506) | <0.0001 | 0.438(0.271-0.709) | 0.0008 |
| Mucinous adenocarcinoma | 0.487(0.276-0.859) | 0.0131 | 0.647(0.361-1.161) | 0.1442 |
| Other | 0.643(0.198-2.09) | 0.4627 | 0.649(0.198-2.135) | 0.4770 |
| **Lymphovascular invasion** |  |  |  |  |
| No |  |  |  |  |
| Yes | 1.733(1.327-2.262) | 0.0001 | 1.356(1.011-1.817) | 0.0417 |
| **MMR status** |  |  |  |  |
| pMMR |  |  |  |  |
| dMMR | 0.613(0.335-1.119) | 0.1111 |  |  |
| **Resection grade** |  |  |  |  |
| R0 |  |  |  |  |
| R1 | 2.29(1.081-4.849) | 0.0305 | 2.171(0.999-4.721) | 0.0504 |
| R2 | 4.202(2.078-8.497) | 0.0001 | 3.71(1.789-7.695) | 0.0004 |
| **Complication** |  |  |  |  |
| No |  |  |  |  |
| Yes | 1.45(1.061-1.982) | 0.0199 | 1.435(1.046-1.969) | 0.0252 |

Parameters with P values <0.05 in the univariate Cox model were then entered into a final multivariable Cox regression model.

CI = confidence interval, PNI = perineural invasion,MMR = mismatch repair.

**Table S5** Univariate and Multivariable Cox models for disease-free survival of patients with stage Ⅲ disease.

|  | **Univariate Cox model** | | **Multivariable Cox model** | |
| --- | --- | --- | --- | --- |
|  | **Hazard Ratio** | **P-value** | **Hazard Ratio** | **P-value** |
|  | **(95% CI)** |  | **(95% CI)** |  |
| **PNI** |  |  |  |  |
| No |  |  |  |  |
| Yes | 1.702(1.375-2.107) | <0.0001 | 1.514(1.211-1.892) | 0.0003 |
| **Gender** |  |  |  |  |
| Female |  |  |  |  |
| Male | 1.162(0.956-1.412) | 0.1317 |  |  |
| **Age** |  |  |  |  |
| ≥50 |  |  |  |  |
| ＜50 | 0.704(0.559-0.886) | 0.0028 | 0.652(0.51-0.834) | 0.0006 |
| **Family history** |  |  |  |  |
| No |  |  |  |  |
| Yes | 0.976(0.562-1.696) | 0.9324 |  |  |
| **Hypertension** |  |  |  |  |
| No |  |  |  |  |
| Yes | 1.393(1.092-1.776) | 0.0076 | 1.185(0.915-1.534) | 0.1992 |
| **Diabetes** |  |  |  |  |
| No |  |  |  |  |
| Yes | 1.596(1.171-2.175) | 0.0031 | 1.454(1.054-2.006) | 0.0224 |
| **BMI** |  |  |  |  |
| ≤24 |  |  |  |  |
| >24 | 0.899(0.731-1.106) | 0.3133 |  |  |
| **CEA** |  |  |  |  |
| ≤5 |  |  |  |  |
| >5 | 1.303(1.074-1.58) | 0.0072 | 1.197(0.981-1.459) | 0.0760 |
| **Tumor location** |  |  |  |  |
| Colon |  |  |  |  |
| Rectum | 1.186(0.974-1.445) | 0.0896 |  |  |
| Other | 1.363(0.927-2.005) | 0.1151 |  |  |
| **T** |  |  |  |  |
| T1/2 |  |  |  |  |
| T3/4 | 2.34(1.537-3.562) | 0.0001 | 1.871(1.218-2.872) | 0.0042 |
| **N** |  |  |  |  |
| N1 |  |  |  |  |
| N2 | 1.903(1.561-2.319) | <0.0001 | 1.586(1.28-1.965) | <0.0001 |
| **Differentiation** |  |  |  |  |
| Poor |  |  |  |  |
| Median | 0.519(0.379-0.711) | <0.0001 | 0.639(0.455-0.897) | 0.0096 |
| Well | 0.402(0.275-0.587) | <0.0001 | 0.555(0.368-0.836) | 0.0048 |
| Mucinous adenocarcinoma | 0.557(0.344-0.903) | 0.0177 | 0.687(0.419-1.125) | 0.1358 |
| Other | 0.622(0.223-1.728) | 0.3621 | 0.647(0.231-1.811) | 0.4069 |
| **Lymphovascular invasion** |  |  |  |  |
| No |  |  |  |  |
| Yes | 1.704(1.361-2.134) | <0.0001 | 1.359(1.065-1.734) | 0.0137 |
| **MMR status** |  |  |  |  |
| pMMR |  |  |  |  |
| dMMR | 0.696(0.434-1.116) | 0.1325 |  |  |
| **Resection grade** |  |  |  |  |
| R0 |  |  |  |  |
| R1 | 1.813(0.937-3.51) | 0.0775 | 1.289(0.651-2.553) | 0.4659 |
| R2 | 2.164(1.074-4.358) | 0.0307 | 1.921(0.941-3.92) | 0.0728 |
| **Complication** |  |  |  |  |
| No |  |  |  |  |
| Yes | 1.595(1.241-2.05) | 0.0003 | 1.626(1.261-2.096) | 0.0002 |

Parameters with P values <0.05 in the univariate Cox model were then entered into a final multivariable Cox regression model.

CI = confidence interval, PNI = perineural invasion,MMR = mismatch repair.

**Table S6** Tables after adding adjuvant chemotherapy.

**Baseline characteristics**

|  | **PNI** | | **Total (N=3485)** | ***P* value** |
| --- | --- | --- | --- | --- |
|  | No(N=3046) | Yes(N=439) |  |  |
| **adjuvant chemotherapy, n(%)** |  |  |  | 0.0084 |
| No | 1682(55.2) | 213(48.5) | 3027(86.9) |  |
| Yes | 1364(44.8) | 226(51.5) | 28(0.8) |  |

**Univariate and multivariate cox models evaluated the effect of perineural invasion on OS and DFS after adding adjuvant chemotherapy.**

|  | **Univariate Cox model** | | **Multivariate Cox model** | |
| --- | --- | --- | --- | --- |
|  | **Hazard Ratio** | **P-value** | **Hazard Ratio** | **P-value** |
|  | **(95% CI)** |  | **(95% CI)** |  |
| **OS: PNI** † |  |  |  |  |
| No |  |  |  |  |
| Yes | 2.322(1.977-2.727) | <0.0001 | 1.302(1.096-1.546) | 0.0026 |
| **DFS: PNI** ‡ |  |  |  |  |
| No |  |  |  |  |
| Yes | 2.495(2.177-2.859) | <0.0001 | 1.405(1.214-1.626) | <0.0001 |
| **OS in stage III CRC: PNI *** |  |  |  |  |
| No |  |  |  |  |
| Yes | 1.354(1.034-1.772) | 0.0274 | 1.175(0.887-1.558) | 0.2614 |
| **DFS in stage III CRC: PNI *** |  |  |  |  |
| No |  |  |  |  |
| Yes | 1.702(1.375-2.107) | <0.0001 | 1.534(1.224-1.922) | 0.0002 |

Parameters with P values <0.05 in the univariate Cox model were then entered into a final multivariable Cox regression model. Entire tables are shown in the supplementary data.

†Adjusted for PNI, sex, age, family history of colorectal cancer, hypertension, diabetes, BMI, CEA, T, N ,M, differentiation, lymphovascular invasion, MMR status, resection grade, complications and adjuvant chemotherapy.

‡Adjusted for PNI, sex, age , hypertension, diabetes, BMI, CEA, tumor location, T, N ,M, differentiation, lymphovascular invasion, MMR status, resection grade, complications and adjuvant chemotherapy.

*****Adjusted for PNI, age, hypertension, diabetes, CEA, T, N, differentiation, lymphovascular invasion, resection grade, complications and adjuvant chemotherapy. .

**Table S7 Univariate and Multivariable cox models for overall survival of baseline characteristics after adding adjuvant chemotherapy.**

|  | **Univariate Cox model** | | **Multivariable Cox model** | |
| --- | --- | --- | --- | --- |
|  | **Hazard Ratio** | **P-value** | **Hazard Ratio** | **P-value** |
|  | **(95% CI)** |  | **(95% CI)** |  |
| **PNI** |  |  |  |  |
| No |  |  |  |  |
| Yes | 2.322(1.977-2.727) | <0.0001 | 1.302(1.096-1.546) | 0.0026 |
| **Gender** |  |  |  |  |
| Female |  |  |  |  |
| Male | 1.27(1.105-1.46) | 0.0008 | 1.174(1.019-1.352) | 0.0260 |
| **Age** |  |  |  |  |
| ≥50 |  |  |  |  |
| ＜50 | 0.523(0.436-0.629) | <0.0001 | 0.505(0.417-0.613) | <0.0001 |
| **Family history** |  |  |  |  |
| No |  |  |  |  |
| Yes | 0.618(0.392-0.974) | 0.0380 | 0.788(0.498-1.248) | 0.3098 |
| **Hypertension** |  |  |  |  |
| No |  |  |  |  |
| Yes | 1.528(1.296-1.801) | <0.0001 | 1.182(0.992-1.407) | 0.0608 |
| **Diabetes** |  |  |  |  |
| No |  |  |  |  |
| Yes | 1.693(1.373-2.087) | <0.0001 | 1.528(1.230-1.898) | 0.0001 |
| **BMI** |  |  |  |  |
| ≤24 |  |  |  |  |
| >24 | 0.791(0.682-0.918) | 0.0020 | 0.803(0.690-0.935) | 0.0047 |
| **CEA** |  |  |  |  |
| ≤5 |  |  |  |  |
| >5 | 2.316(2.028-2.645) | <0.0001 | 1.285(1.112-1.485) | 0.0007 |
| **Tumor location** |  |  |  |  |
| Colon |  |  |  |  |
| Rectum | 0.889(0.774-1.021) | 0.0950 |  |  |
| Other | 1.206(0.928-1.568) | 0.1614 |  |  |
| **T** |  |  |  |  |
| T1/2 |  |  |  |  |
| T3/4 | 3.032(2.386-3.853) | <0.0001 | 1.663(1.291-2.143) | 0.0001 |
| **N** |  |  |  |  |
| N0 |  |  |  |  |
| N1 | 2.394(2.059-2.783) | <0.0001 | 1.679(1.425-1.979) | <0.0001 |
| N2 | 3.961(3.318-4.728) | <0.0001 | 2.117(1.727-2.594) | <0.0001 |
| **M** |  |  |  |  |
| M0 |  |  |  |  |
| M1 | 6.242(5.438-7.166) | <0.0001 | 1.979(1.541-2.543) | <0.0001 |
| **Stage** |  |  |  |  |
| I |  |  |  |  |
| II | 1.603(1.181-2.176) | 0.0025 |  |  |
| III | 3.216(2.394-4.32) | <0.0001 |  |  |
| IV | 12.746(9.508-17.087) | <0.0001 |  |  |
| **Differentiation** |  |  |  |  |
| Poor |  |  |  |  |
| Median | 0.424(0.335-0.536) | <0.0001 | 0.502(0.390-0.645) | <0.0001 |
| Well | 0.275(0.21-0.359) | <0.0001 | 0.428(0.321-0.572) | <0.0001 |
| Mucinous adenocarcinoma | 0.443(0.31-0.635) | <0.0001 | 0.564(0.391-0.814) | 0.0022 |
| Other | 0.764(0.333-1.752) | 0.5248 | 0.647(0.280-1.497) | 0.3090 |
| **Lymphovascular invasion** |  |  |  |  |
| No |  |  |  |  |
| Yes | 2.463(2.079-2.919) | <0.0001 | 1.315(1.089-1.588) | 0.0045 |
| **MMR status** |  |  |  |  |
| pMMR |  |  |  |  |
| dMMR | 0.45(0.321-0.63) | <0.0001 | 0.598(0.422-0.847) | 0.0038 |
| **Resection grade** |  |  |  |  |
| R0 |  |  |  |  |
| R1 | 2.508(1.342-4.687) | 0.0040 | 1.901(1.010-3.579) | 0.0466 |
| R2 | 7.234(6.271-8.345) | <0.0001 | 2.648(2.053-3.416) | <0.0001 |
| **Complication** |  |  |  |  |
| No |  |  |  |  |
| Yes | 1.786(1.506-2.119) | <0.0001 | 1.699(1.429-2.019) | <0.0001 |
| **Adjuvant chemotherapy n(%)** |  |  |  |  |
| No |  |  |  |  |
| Yes | 0.747(0.653-0.855) | <0.0001 | 0.702(0.609-0.809) | <0.0001 |

Parameters with P values <0.05 in the univariate Cox model were then entered into a final multivariable Cox regression model.

CI = confidence interval, PNI = perineural invasion,MMR = mismatch repair.

**Table S8 Univariate and Multivariable cox models for disease-free survival of baseline characteristics after adding adjuvant chemotherapy.**

|  | **Univariate Cox model** | | **Multivariable Cox model** | |
| --- | --- | --- | --- | --- |
|  | **Hazard Ratio** | **P-value** | **Hazard Ratio** | **P-value** |
|  | **(95% CI)** |  | **(95% CI)** |  |
| **PNI** |  |  |  |  |
| No |  |  |  |  |
| Yes | 2.495(2.177-2.859) | <0.0001 | 1.405(1.214-1.626) | <0.0001 |
| **Gender** |  |  |  |  |
| Female |  |  |  |  |
| Male | 1.182(1.051-1.329) | 0.0051 | 1.077(0.956-1.213) | 0.2197 |
| **Age** |  |  |  |  |
| ≥50 |  |  |  |  |
| ＜50 | 0.756(0.657-0.869) | 0.0001 | 0.704(0.607-0.815) | <0.0001 |
| **Family history** |  |  |  |  |
| No |  |  |  |  |
| Yes | 0.792(0.56-1.119) | 0.1867 |  |  |
| **Hypertension** |  |  |  |  |
| No |  |  |  |  |
| Yes | 1.344(1.164-1.553) | 0.0001 | 1.172(1.005-1.367) | 0.0436 |
| **Diabetes** |  |  |  |  |
| No |  |  |  |  |
| Yes | 1.449(1.201-1.747) | 0.0001 | 1.445(1.190-1.755) | 0.0002 |
| **BMI** |  |  |  |  |
| ≤24 |  |  |  |  |
| >24 | 0.848(0.749-0.96) | 0.0092 | 0.862(0.759-0.978) | 0.0210 |
| **CEA** |  |  |  |  |
| ≤5 |  |  |  |  |
| >5 | 2.033(1.817-2.275) | <0.0001 | 1.253(1.109-1.415) | 0.0003 |
| **Tumor location** |  |  |  |  |
| Colon |  |  |  |  |
| Rectum | 0.994(0.885-1.117) | 0.9207 | 1.116(0.988-1.262) | 0.0781 |
| Other | 1.364(1.095-1.699) | 0.0056 | 1.275(1.021-1.594) | 0.0323 |
| **T** |  |  |  |  |
| T1/2 |  |  |  |  |
| T3/4 | 2.509(2.083-3.021) | <0.0001 | 1.436(1.176-1.752) | 0.0004 |
| **N** |  |  |  |  |
| N0 |  |  |  |  |
| N1 | 2.302(2.028-2.613) | <0.0001 | 1.515(1.322-1.736) | <0.0001 |
| N2 | 3.912(3.364-4.549) | <0.0001 | 2.019(1.703-2.395) | <0.0001 |
| **M** |  |  |  |  |
| M0 |  |  |  |  |
| M1 | 5.068(4.49-5.722) | <0.0001 | 2.831(2.314-3.462) | <0.0001 |
| **Stage** |  |  |  |  |
| I |  |  |  |  |
| II | 1.385(1.095-1.752) | 0.0067 |  |  |
| III | 2.88(2.297-3.612) | <0.0001 |  |  |
| IV | 9.174(7.298-11.532) | <0.0001 |  |  |
| **Differentiation** |  |  |  |  |
| Poor |  |  |  |  |
| Median | 0.507(0.41-0.627) | <0.0001 | 0.664(0.531-0.831) | 0.0003 |
| Well | 0.325(0.256-0.413) | <0.0001 | 0.571(0.443-0.736) | <0.0001 |
| Mucinous adenocarcinoma | 0.527(0.385-0.72) | 0.0001 | 0.703(0.511-0.966) | 0.0300 |
| Other | 1.067(0.556-2.047) | 0.8460 | 0.895(0.463-1.731) | 0.7410 |
| **Lymphovascular invasion** |  |  |  |  |
| No |  |  |  |  |
| Yes | 2.224(1.914-2.585) | <0.0001 | 1.180(1.001-1.390) | 0.0487 |
| **MMR status** |  |  |  |  |
| pMMR |  |  |  |  |
| dMMR | 0.444(0.334-0.591) | <0.0001 | 0.614(0.457-0.823) | 0.0011 |
| **Resection grade** |  |  |  |  |
| R0 |  |  |  |  |
| R1 | 2.496(1.522-4.093) | 0.0003 | 1.849(1.118-3.058) | 0.0167 |
| R2 | 4.437(3.898-5.051) | <0.0001 | 1.318(1.064-1.633) | 0.0114 |
| **Complication** |  |  |  |  |
| No |  |  |  |  |
| Yes | 1.675(1.445-1.942) | <0.0001 | 1.47(1.265-1.709) | <0.0001 |
| **Adjuvant chemotherapy n(%)** |  |  |  |  |
| No |  |  |  |  |
| Yes | 1.276(1.141-1.428) | <0.0001 | 1.284(1.139-1.448) | <0.0001 |

Parameters with P values <0.05 in the univariate Cox model were then entered into a final multivariable Cox regression model.

CI = confidence interval, PNI = perineural invasion,MMR = mismatch repair.
